# Calculating Bias in Test Score Equating in a NEAT Design

**DOI:** 10.1177/01466216251330305

**Published:** 2025-03-24

**Authors:** Marie Wiberg, Inga Laukaityte

**Affiliations:** 1 8075 Umeå University, Sweden

**Keywords:** criterion function, frequency estimation, chained equating

## Abstract

Test score equating is used to make scores from different test forms comparable, even when groups differ in ability. In practice, the non-equivalent group with anchor test (NEAT) design is commonly used. The overall aim was to compare the amount of bias under different conditions when using either chained equating or frequency estimation with five different criterion functions: the identity function, linear equating, equipercentile, chained equating and frequency estimation. We used real test data from a multiple-choice binary scored college admissions test to illustrate that the choice of criterion function matter. Further, we simulated data in line with the empirical data to examine difference in ability between groups, difference in item difficulty, difference in anchor test form and regular test form length, difference in correlations between anchor test form and regular test forms, and different sample size. The results indicate that how bias is defined heavily affects the conclusions we draw about which equating method is to be preferred in different scenarios. Practical implications of this in standardized tests are given together with recommendations on how to calculate bias when evaluating equating transformations.

## Introduction

Test score equating is a procedure in which statistical models are used to place scores from different test forms on the same score scale (González & Wiberg, 2017). Equating is important when either the test forms differ or the ability levels of the groups taking the different test forms differ. If the groups that take the test forms can be assumed to be similar, equivalent groups (EG) design can be used. If the groups cannot be assumed to be similar, the non-equivalent groups with anchor test (NEAT) design can be used instead, provided that a set of common items (i.e., an anchor test) is given to the groups that take the different test forms. If different equating methods are used, we should evaluate and compare the equating transformations to select the most suitable method. The evaluation can be done with several different measures, and different aspects need to be examined depending on whether the compared methods are from the same or different equating frameworks (Leôncio et al., 2022; Wiberg & González, 2016). Harris and Crouse (1983) thoroughly described how to evaluate equating transformations using different criteria. One evaluation measure they mentioned was bias, which is the focus of this article. Bias have been used in several equating studies (e.g., van der Linden, 2006; Wiberg et al., 2014; Wallmark et al., 2023; Wallin & Wiberg, 2023). To calculate bias, let ${\hat{\varphi }}_{Y}\left(x\right)$ denote the estimated equating transformation when equating test form X to test form Y and let $\varphi_{Y}\left(x\right)$ denote the true equating transformation. If E is the expected value, then, the bias is formally defined as

(1) $$\text{Bias}\left({\hat{\varphi }}_{Y}\left(x\right)\right)=E\left[{\hat{\varphi }}_{Y}\left(x\right)-\varphi_{Y}\left(x\right)\right].$$

The equating transformation depends on the used data collection design and the chosen equating method, and here the focus is on two equating methods in the NEAT design. The challenge when calculating bias is how to define the true equating transformation. Equating errors are many times defined for a fixed criterion equating function that specifies the true equated score for each number-correct score in a scale. For a review of different criterion equating functions, refer to Kolen and Brennan (2014, Sect. 8.4), who describe different options depending on data collection design, sample size or if simulations are used. They summarized four equating criteria: error in estimating equating relationships, equating in a circle, group invariance, and equity property. Our study focuses on errors in estimating equating relationships, in which one can use pseudo test forms, pseudo groups, a single group criterion or a model-based criterion.

In previous research, several different criterion equating functions have been used. For example, Kim et al. (2020) used three different criteria for the equating relationship. First, they established the criterion equating relationships based on Kim and Lee (2016) proposal of using a large-sample single-group equipercentile equating. Secondly, they used the identity equating and thirdly, they used equipercentile equating based on the entire sample who took the examined test form. Albano & Wiberg (2019) used equipercentile equating as a true equating transformation criterion in the NEAT design. In van der Linden (2006) and Wiberg et al. (2014), the true equating transformation was a model-based family of equating transformations. In Wallmark et al. (2023) and Wallin and Wiberg (2023), the true equating transformation used was a model-based criterion obtained using replicates in their simulation studies. Further, in Wiberg and González (2016), an equating transformation from one equating method was used as the true equating transformation, and in Leoncio et al. (2022) two approaches were used, for real data they followed Lord (1980, p. 203) who equated the test to itself (i.e., identity equating criterion) and for simulated data they used true item parameters to generate the true equating transformation (i.e., model-based criterion).

The choice of criterion function when calculating bias also depends on the chosen equating method. When using the NEAT design, one can either perform the equating with frequency estimation (FE) or chained equating (CE). These methods give in general similar equating results although CE tend to work better when groups have different abilities (see e.g., Eignor et al., 1990; Harris & Kolen, 1990; Lawrence & Dorans, 1990; von Davier et al., 2004). In the past, FE has been found to produce more bias than CE when group differences are large (e.g., Powers & Kolen, 2014; Wang et al., 2008). These studies, however, only used one criterion function when calculating bias. For example, Wang et al. (2008), used a NEAT design with an internal anchor test form when examining linear equating methods and used the average over replicates of the equating transformation in the simulation study as the true equating transformation (i.e., model-based criterion). Further, Kim et al. (2008) used chained linear equating as a criterion function when calculating bias when examining small-sample equating. To the best of our knowledge, there are not yet any studies of bias in equating when several criterion functions are compared when we have a NEAT design. The overall aim was to compare the amount of bias under different conditions when using either CE or FE with different criterion functions. Real empirical test data from a college admissions test was used to illustrate that the choice of criterion function matters when calculating the bias. A simulation study was conducted to examine different conditions including the impact of higher ability in one of the groups, more difficult test form, different correlations between anchor test form and regular test form, different sample size, different test length of the anchor test form and the regular test form. This research is important as failing to establish relevant evaluation criteria, including the fair selection of a criterion function, risks leading to incorrect conclusions about the performance of methods when comparing them. Note, the goal here is not to identify the best criterion function for a specific situation but rather to illustrate that the choice of criterion function has an impact when calculating bias in different contexts.

The rest of this article is structured as follows. In the next section, the equating methods and criterion functions used are briefly described, followed by a description of the bias calculation. Subsequently, an empirical study is presented in which bias is calculated with five different criterion functions, followed by a simulation study examining several different scenarios. The article ends with a discussion, which includes some final remarks and a practical recommendation.

## Test Score Equating Methods and Criterion Functions

In this article, we focus on the case when we have a NEAT design, that is, different populations are given different regular test forms and a common anchor test form. Throughout this article, we assume that the anchor test form is external. Assume that we have a new test form X with test scores *X* and an old test form Y with test scores *Y*. The test scores are random variables from the populations *P* and *Q*, respectively. Assume further that *X* and *Y* are continuous, and we denote their cumulative density functions (CDFs) with $F_{X}\left(x\right)$ and $F_{Y}\left(y\right)$ , respectively. A formal definition of an equivalent test score *y* on test form Y for a test score *x* on test form X is the equipercentile equating transformation (see e.g., Kolen & Brennan, 2014), defined as

(2) $$\varphi_{Y}\left(x\right)=F_{Y}^{-1}\left(F_{X}\left(x\right)\right).$$

### Frequency Estimation

For an EG design, frequency estimation (FE) equipercentile equating (Angoff, 1971; Braun & Holland, 1982) can be directly obtained from equation (2). For a NEAT design with an anchor test form A with scores *a*, we need to construct CDFs built on the joint probabilities of the target population. Define a synthetic target population *T*, as T=wP+(1−w)Q , where w≤1 , and *T* is a mixture of populations *P* and *Q*. Next, assume that (i) the conditional distribution of total scores on test form X for a given score point in A is the same across populations, and (ii) the conditional distribution of total scores on test form Y for a given score point in A is the same across populations. The synthetic distributions can then be obtained by using:

(3) $$f_{XT}\left(x\right)=wf_{P}\left(x\right)+\left(1-w\right)\sum f_{P}\left(x|a\right)f_{Q}\left(a\right),\mathrm{and}\;f_{YT}\left(y\right)=\left(1-w\right)f_{Q}\left(y\right)+w\sum f_{Q}\left(y|a\right)f_{P}\left(a\right),$$

where f(x) , f(y) , and f(a) are the density functions for test forms X, Y, and A, respectively, in the populations *P* and *Q* as defined in the index and f(x|a) , f(y|a) are the conditional density functions of *x* and *y* given *a*, respectively. By cumulating the distributions in (3) over the score values, we obtain the corresponding CDFs $F_{XT}\left(x\right)$ and $F_{YT}\left(y\right)$ which can be plugged into equation (2), to obtain the FE equating transformation as

(4) $$\varphi_{Y}\left(x\right)=F_{YT}^{-1}\left(F_{XT}\left(x\right)\right).$$

This equating transformation will be used both as an equating method and a criterion function. Frequency estimation has been used as a criterion function in the NEAT design by, for example, Albano (2016).

#### Chained equating

Chained equating (CE), introduced by Angoff (1971) and named by Dorans (1990) and Livingston et al., (1990) is obtained by linking the CDFs of test forms X and Y through the anchor test form CDFs $H_{AX}$ and $H_{AY}$ in population *P* and *Q* respectively. If we use equation (2) several times, we can define the CE transformation as

(5) $$\varphi_{Y}\left(x\right)=F_{Y}^{-1}\left(H_{AY}\left(H_{AX}^{-1}\left(F_{X}\left(x\right)\right)\right)\right).$$

This equating transformation will be used both as an equating method and a criterion function. Chained equating has been used as a criterion function in the NEAT design by, for example, Albano (2016).

#### Equipercentile equating

In addition, to the criterion functions from the NEAT design (i.e., CE and FE), we also include the possibility to use the equipercentile equating transformation in the EG design, which means to use equation (2) directly, see e.g., Kolen and Brennan (2014). Equipercentile equating has been used as a criterion function by, for example, Oh and Moses (2012), Albano & Wiberg (2019), and Wang et al. (2020) in the NEAT design.

#### Linear equating

Linear equating is another equating criterion function in the EG design that was used in this study. The general linear equating transformation is defined as

(6) $$\varphi_{Y}\left(x\right)=\mu \left(Y\right)+\frac{\sigma \left(Y\right)}{\sigma \left(X\right)}\left(x-\mu \left(X\right)\right),$$

where σ(X) and σ(Y) are the standard deviations, and μ(X) and μ(Y) are the means (Kolen & Brennan, 2014). Linear equating as a criterion function has been used previously by, for example, Liu et al. (2011) with the NEAT design.

#### Identity equating

Finally, we have included the possibility to use identity equating as a criterion function, which was first described by Lord (1980, p. 203) when equating test scores, and have been used by several researchers for equating test scores (e.g., Kim et al., 2011; Moses et al., 2007; Almond, 2014). Identity equating considers the identity function as a true form of equating, where a score on form Y is directly matched to a score on form X without requiring any additional transformation. Identity equating has been used as a criterion function when calculating absolute bias and root mean squared error in, for example, Wang et al. (2020) and Kim et al. (2020) in the NEAT design.

## Calculating Bias

From the general definition of bias in equation (1), the bias can be calculated for each score value as

(7) $$\text{Bias}\left[{\hat{\varphi }}_{Y}\left(x_{i}\right)\right]={\hat{\varphi }}_{Y}\left(x_{i}\right)-\varphi_{Y}\left(x_{i}\right),$$

where is ${\hat{\varphi }}_{Y}\left(x_{i}\right)$ is the estimated equated value in score $x_{i}$ , and $\varphi_{Y}\left(x_{i}\right)$ is the true equated value in score $x_{i}$ . The choice of criterion function to obtain $\varphi_{Y}\left(x_{i}\right)$ is crucial for calculating bias of the equating transformation.

In this article, the five previously defined criterion functions for the true equating transformations were used: i) the FE transformation defined in equation (4), and labeled fe, ii) the CE transformation defined in equation (5), and labeled ce, iii) the equipercentile equating transformation as defined for equivalent groups in equation (2) and labeled eq, iv) the general linear equating transformation defined for the EG design in equation (6) and labeled li, and v) the identity equating transformation defined when we have an EG design and labeled id.

## Empirical Study

In the empirical study, data from two administrations of the college admissions test, Swedish Scholastic Aptitude Test (SweSAT), were used. The SweSAT is typically given twice a year and contains 160 binary scored multiple-choice items divided into a verbal section and a quantitative section, each comprising 80 items and these sections are equated separately. Each section is administered to the test takers as two booklets with 40 items. The test takers also receive an extra booklet of 40 items which can be either verbal or quantitative, and this booklet contains either tryout items or an external anchor test form of either verbal or quantitative content. The test takers are unaware which booklets are regular booklets, and which booklet is either tryout items or an external anchor test form. In summary, the test takers receive a total of 200 items distributed equally in five booklets. Two regular SweSAT verbal test forms (2015A and 2013A) containing 80 items each, and one 40-item external verbal anchor test form (labeled V) were used. Although the SweSAT are typically administered to between 40,000 and 75,000 test takers, less than 2000 test takers receive an anchor test form due to test security. In the empirical study, we used the NEAT design for the test takers who received the anchor test form and the EG design for the full samples who were administered the different SweSAT test forms.

The test forms were examined with equated values, bias and descriptive statistics, such as mean, standard deviations and correlation measures. The R package *equate* (Albano, 2016) was used to perform the equating and to calculate bias and standard errors. The bias and standard errors were obtained by using the *equate* bootstrap procedure with 1000 replications. The bootstrap procedure works as follows. Samples of sizes *x*_*n*_ and *y*_*n*_ are randomly drawn, with replacement, from each score distribution. Y-equivalent values for each test form X score are then generated using either the equating output or the provided arguments. Standard errors are computed as the standard deviations across the replications for each score point. Bias is calculated as the average equated score across replications, minus the criterion (Albano, 2016). Note, we are aware that using a real data example has large limitations, as the true equating relationship is not known, and thus, we cannot evaluate the methods properly. The primary aim of including the empirical study was to illustrate that the choice of criterion function matters. However, a thorough examination of different criterion functions in various situations is deferred to the subsequent simulation study.

## Results of the Empirical Study

In Table 1, the sample sizes, means, standard deviation (SD) and correlations between the regular verbal test forms and the anchor test form are given. The first four lines have smaller samples as those are the sample sizes that received the anchor test form. The last two lines is the full sample from the whole Sweden. Note that the mean in the anchor test group of the regular test form is higher than the mean for the full sample in 2013 and less than the full sample in 2015. This is because the anchor test form was distributed in 2013 to a town in which the SweSAT results tend to be higher than those from the town that received the anchor test form in 2015, see Laukaityte and Wiberg (2024), who concluded that the location where the anchor test form is distributed may affect the equating results. Also, note that the correlations were high (0.85 and 0.86) between the regular and anchor test forms at both administrations.

**Table 1.** Descriptive Statistics of the two Verbal Regular test Forms and the Anchor test Form.

Test form	Sample size	Items	Mean	SD	Correlation
2013A	1657	80	45.74	12.48	0.86
2013 anchor V	1657	40	20.75	7.39	
2015A	2099	80	42.01	13.38	0.85
2015 anchor V	2099	40	17.45	7.00	
2013A (full group)	59,475	80	42.71	12.80	
2015A (full group)	74,437	80	44.34	13.44	

SD = Standard deviation.

**Figure 1.**
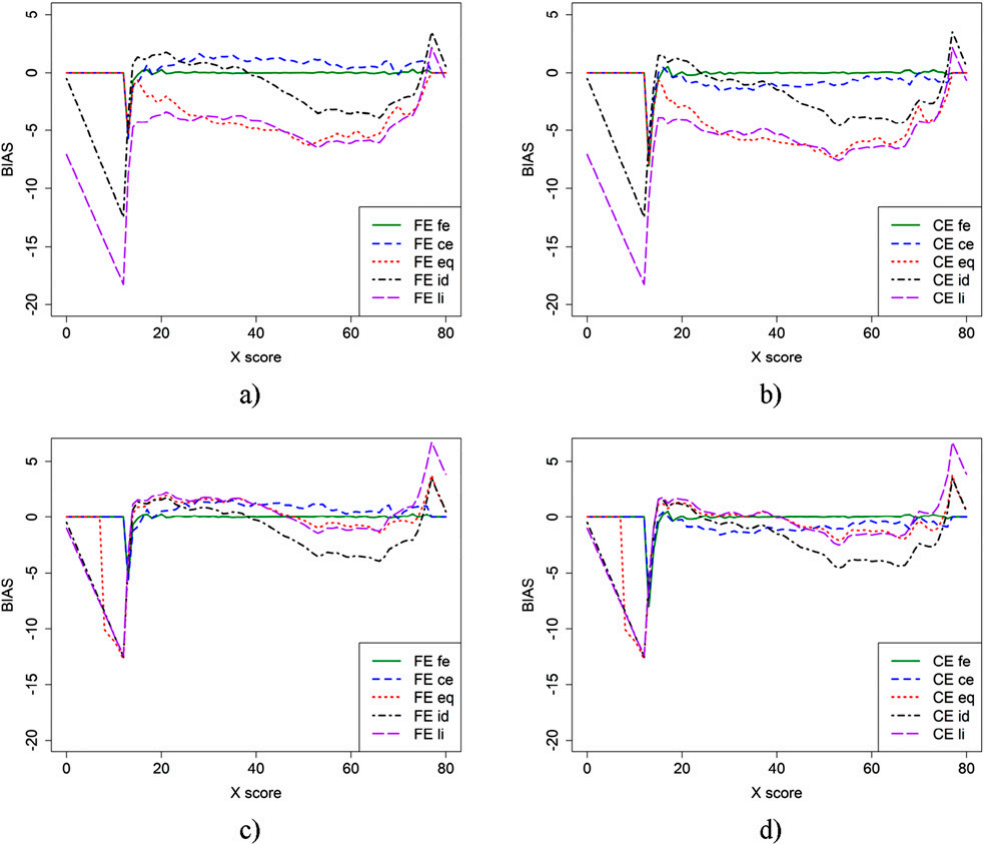
Bias for CE and FE with five different criterion functions when groups contain only those who took anchor test form (a and b) or when full groups were used for eq, li, and id equating (c and d).

In Figure 1, the bias is presented for the two equating methods (FE and CE) using different criterion functions and two sample sizes for the EG design. The top panel (a and b) is based on the anchor test form samples, while the bottom panel (c and d) includes both the anchor test form samples for fe and ce, as well as the full samples for the remaining criterion functions. From Figure 1, we observe that using the full sample instead of just the anchor sample resulted in lower bias when the li and eq criterion functions were applied. The fe criterion function produced very low bias regardless of the equating method used. The CE criterion function, however, resulted in varying bias functions depending on which equating method was used.

## Simulation Study

A simulation study was conducted to be able to examine bias when different criterion functions are used under different conditions. The simulation study was set up to mirror the empirical study, and thus, we used 80 regular items in each test form and 40 external anchor items. The three-parameter logistic item response theory model was used to generate the test scores, with item parameters obtained from the empirical data. The following item parameters for both the regular test forms and the anchor test forms were used: item discrimination *a*∼ LogNormal(0.3,0.4), item difficulty *b* ∼ N(0.4,1) and item guessing *c* ∼ Beta(1.6,6), which are the same as in Laukaityte and Wiberg (2024). The correlations between the regular test forms and the anchor test form varied from 0.78 to 0.85 (except in cases where correlation was intentionally set low), which is like the real empirical data.

We used 2000 test takers for each test form X and Y, and we assumed in the baseline case that the groups who took both test forms had similar ability N(0,1) distribution. Bias was examined when varying the following: if one test form was more difficult (+0.5 was added to the item difficulty of the test form), if one group was more able (the ability distribution was set to N(0.5,1) for one of the groups), if the anchor test form was shortened (from 40 items to 30 items), if both the anchor test form and the regular test forms were much shorter (20 items and 40 items, respectively), if there was a lower correlation between the anchor test form and the regular test forms (0.7 and 0.5), and a smaller sample size (1000 and 500 test takers). The shorter anchor test length was guided by previous equating research studies, which indicate that the length of the anchor test form typically ranges from 20 to 60 items (e.g., Kolen & Brennan, 2014, p. 271; Petersen et al., 1982; von Davier et al., 2004, p. 156). Note, Fitzpatrick (2008) warns explicitly against anchor tests with fewer than 15 items. The smaller sample sizes were included as we wanted to examine impact of fewer test takers. In total, we examined twenty-two scenarios, summarized in Table 2, and each scenario was repeated 500 times.

**Table 2.** Examined scenarios (S1-S22) in the Simulation Study Together With Correlations (C_XA_ and C_YA_) Between the Regular test Forms and the Anchor test Form.

Scenario	*P=Q*	*P*	Y_b_	X_b_	A_b_	N	Items R	Items A	C_XA_	C_YA_
**S1**	X					2000	80	40	0.84	0.83
**S2**		+				2000	80	40	0.85	0.83
**S3**	X		+			2000	80	40	0.84	0.82
**S4**		+	+			2000	80	40	0.85	0.82
**S5**	X			+		2000	80	40	0.84	0.83
**S6**		+		+		2000	80	40	0.84	0.83
**S7**	X				+	2000	80	40	0.83	0.81
**S8**		+			+	2000	80	40	0.84	0.81
**S9**	X		+	+	+	2000	80	40	0.83	0.80
**S10**		+	+	+	+	2000	80	40	0.84	0.80
**S11**	X					2000	80	30	0.83	0.81
**S12**		+				2000	80	30	0.83	0.81
**S13**	X					1000	80	40	0.84	0.83
**S14**		+				1000	80	40	0.85	0.83
**S15**	X					500	80	40	0.84	0.83
**S16**		+				500	80	40	0.85	0.83
**S17**	X					2000	40	20	0.78	0.75
**S18**		+				2000	40	20	0.81	0.75
**S19**	X					2000	80	40	0.71	0.69
**S20**		+				2000	80	40	0.71	0.69
**S21**	X					2000	80	40	0.53	0.52
**S22**		+				2000	80	40	0.53	0.52

P = Q Populations have the same ability distribution, P = Population P is more able, Y_b_ = More difficult Y test, X_b_ = more difficult X test, A_b_ = Anchor test form is more difficult, N = Sample size, ItemsR = Number of items in the regular test form, ItemsA = Number of items in the anchor test form, C_XA_ = Correlation between test form X and anchor test form A, and C_YA_ = Correlation between test form Y and anchor test form A.

We estimated bias using the five previously described criterion functions. As in the empirical study, we used the R package *equate* (Albano, 2016) and the code can be found on the following github https://github.com/inla-files/BiasArticle. Omitted figures in the simulation study can also be found on that github. Bias for each simulation replication was calculated in the same way as in the empirical study, using the bootstrap procedure in the *equate* R package. However, only 100 bootstrap replications were used in the simulation study due to the lengthy calculation time. The final bias was computed as the average over the 500 replicates.

To summarize the differences across all the score points, we calculated the weighted absolute bias (WAB: Liu et al., 2011) defined as

$$\text{WAB}\left[{\hat{\varphi }}_{Y}\left(x\right)\right]=\frac{1}{N}\sum f_{x_{i}}\left|\text{Bias}\left[{\hat{\varphi }}_{Y}\left(x_{i}\right)\right]\right|,$$

where $f_{x_{i}}$ is the frequency at a particular score $x_{i}$ in the new test form sample and *N* is the number of test takers who received the new test form. For all scenarios, N was set to 2,000, except for scenarios S13-S14, where N was set to 1,000, and S15-S16, where N was set to 500.

## Results from the Simulation Study

**Figure 2.**
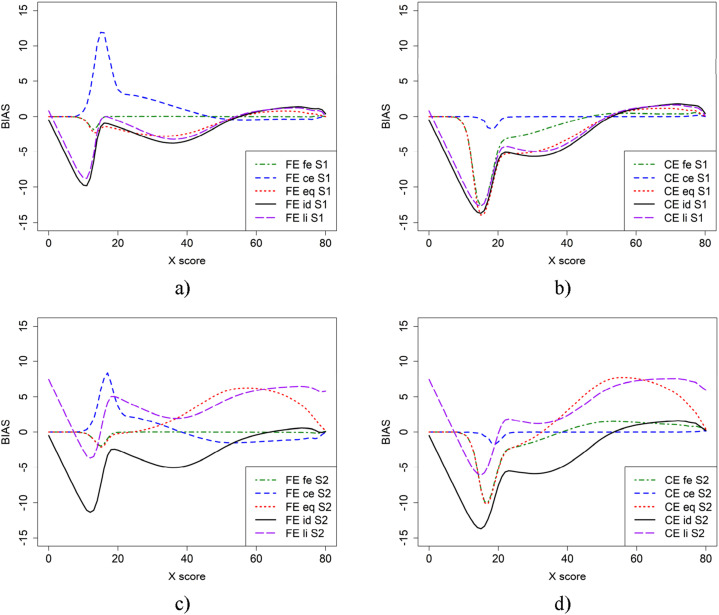
Bias in the baseline scenario 1 (a and b) and scenario 2 (c and d) for CE and FE with five different criterion functions.

Figure 2 illustrates the bias in scenario 1 (baseline) and scenario 2, where more able test takers are in population *P*, using CE and FE with the five criterion functions. From Figure 2, it is evident that the choice of criterion function heavily affects the conclusions we draw about bias. When the criterion function is the same as the equating method used, the bias is small for both CE and FE. However, when the criterion function and the equating method are not the same, the bias is large for lower scores. For scenario 1 (Figures 2(a) and 2(b)), when groups are of similar abilities, criterion functions id and li for both CE and FE yielded larger bias on the lower score scale compared to fe for FE and ce for CE. The eq criterion function yielded lower bias compared to id and li, which were very similar. However, the difference is much smaller for CE equating than for FE.

For scenario 2 (Figure 2(c) and 2(d)), where groups are of differing abilities, both eq and li criterion functions resulted in visually different bias, while id remained the same. In this case, the eq and li criterion functions yield higher bias on the upper score scale. Furthermore, there was a large difference between the id and li criterion functions. Note that the dip before score 20 is likely due to few test takers in that score range.

**Figure 3.**
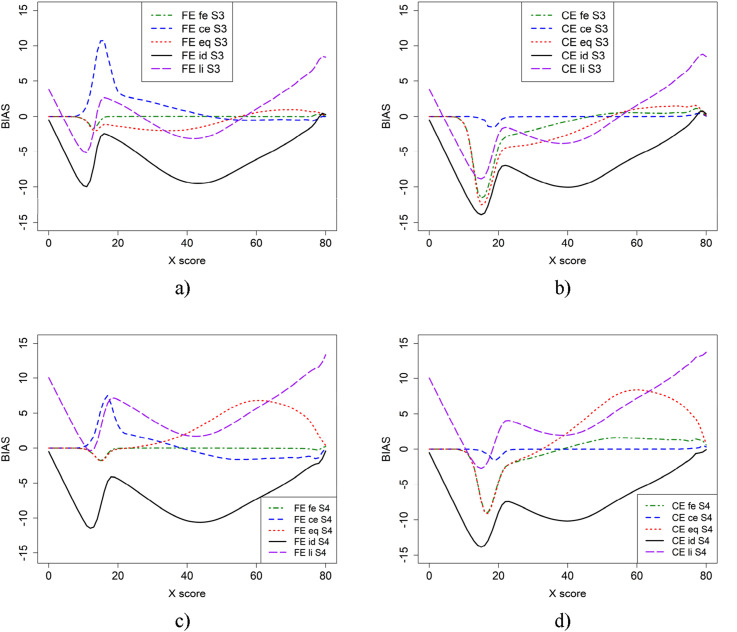
Scenario 3 (a and b) and 4 (c and d), which is similar to scenario 1 and 2 except that test form Y (b + 0.5) is more difficult.

Scenarios 3 and 4 are like scenarios 1 and 2, but with test form Y more difficult and are displayed in Figure 3. The largest differences between Figures 2 and 3 occurred when id was used as the criterion function, in which case the bias was much larger. The difference between bias functions when id and li were used as criterion functions are also larger than in scenarios 1 and 2.

**Figure 4.**
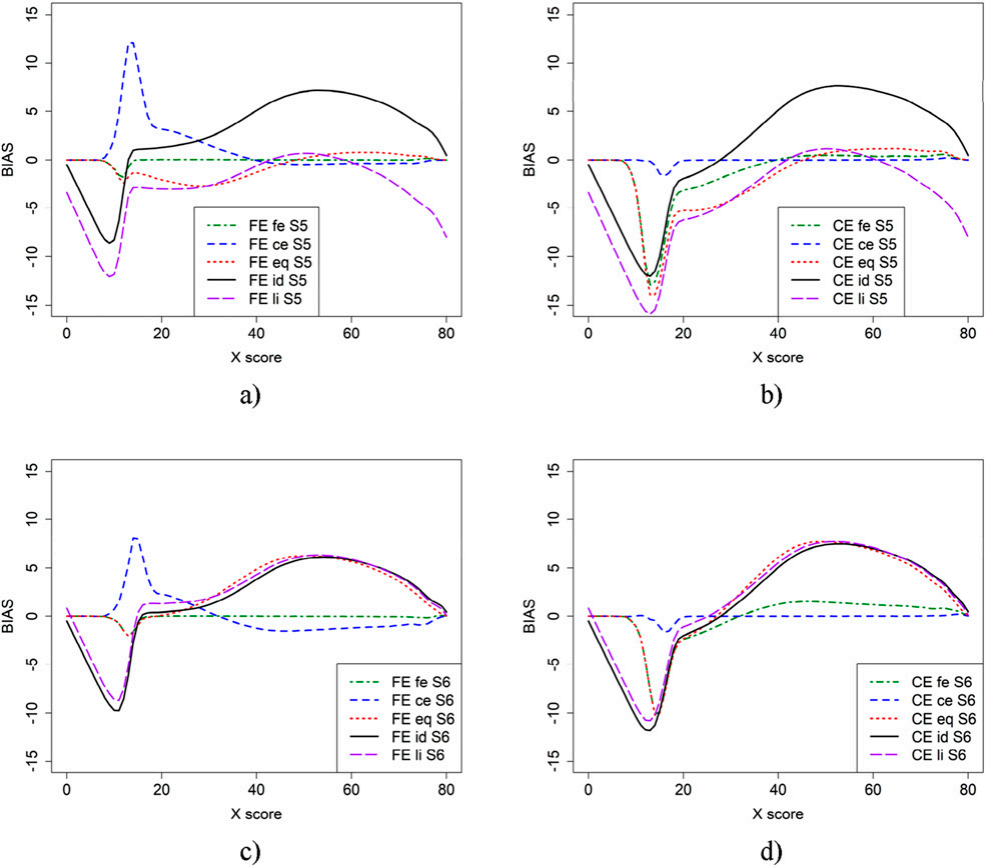
Scenario 5 (a and b) and 6 (c and d), which is similar to scenario 1 and 2 but test form X (b + 0.5) is more difficult.

In Figure 4, bias results for the scenarios where test form X is more difficult than in the baseline case (scenario 5) and when population *P* is more able (scenario 6) are presented. In general, using id and li as criterion functions yields larger bias for both FE and CE. Note that, in contrast to scenarios 1 and 2, there was almost no difference between the bias functions for id an li when the groups were of differing abilities (Figures 4(c) and 4(d)), and a large difference when the groups were of similar ability (Figures 4(a) and 4(b)).

We also examined the impact on bias of a more difficult anchor test form for populations with similar abilities (Scenario 7) and when population *P* was more able (Scenario 8). Overall, the bias is very similar to the bias results in Figure 2, so we have omitted the figures, but they can be found on the provided github. We further studied scenarios where both the regular test forms and the anchor test form were more difficult for populations with similar abilities (Scenario 9) and when population *P* was more able (Scenario 10). As the resulting plots are like the plots in Figure 2, we have omitted the figures, but they can be found on the provided github.

**Figure 5.**
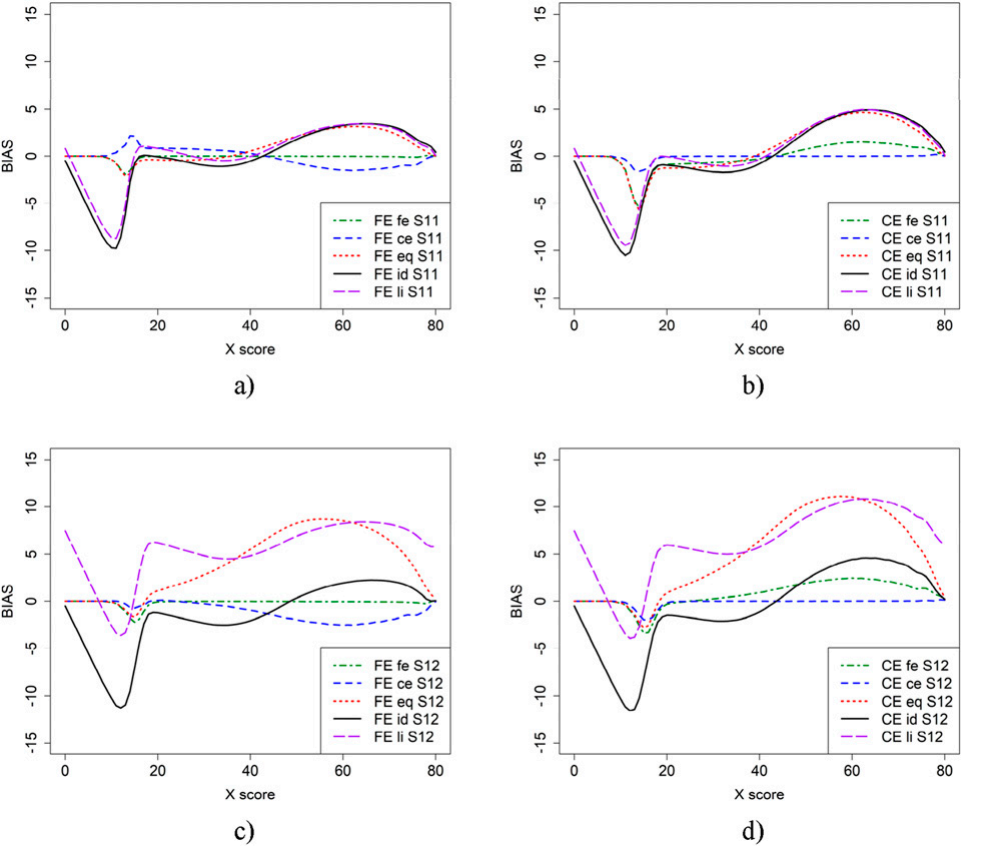
Scenario 11 (a and b) and 12 (c and d), which is similar to scenario 1 and 2 but shorter anchor (30 items) test form.

To examine the impact of anchor test length on equating, we repeated Scenarios 1 and 2 from Figure 2, but with a shorter anchor test containing 30 items in Figure 5. This was done for populations with similar abilities (Scenario 11) and populations with differing abilities (Scenario 12). The largest difference in bias appeared for FE at low scores when ce was used as the criterion function, compared to the previous scenarios. However, the bias with criterion functions eq, id, and li changed only slightly in both scenarios.

**Figure 6.**
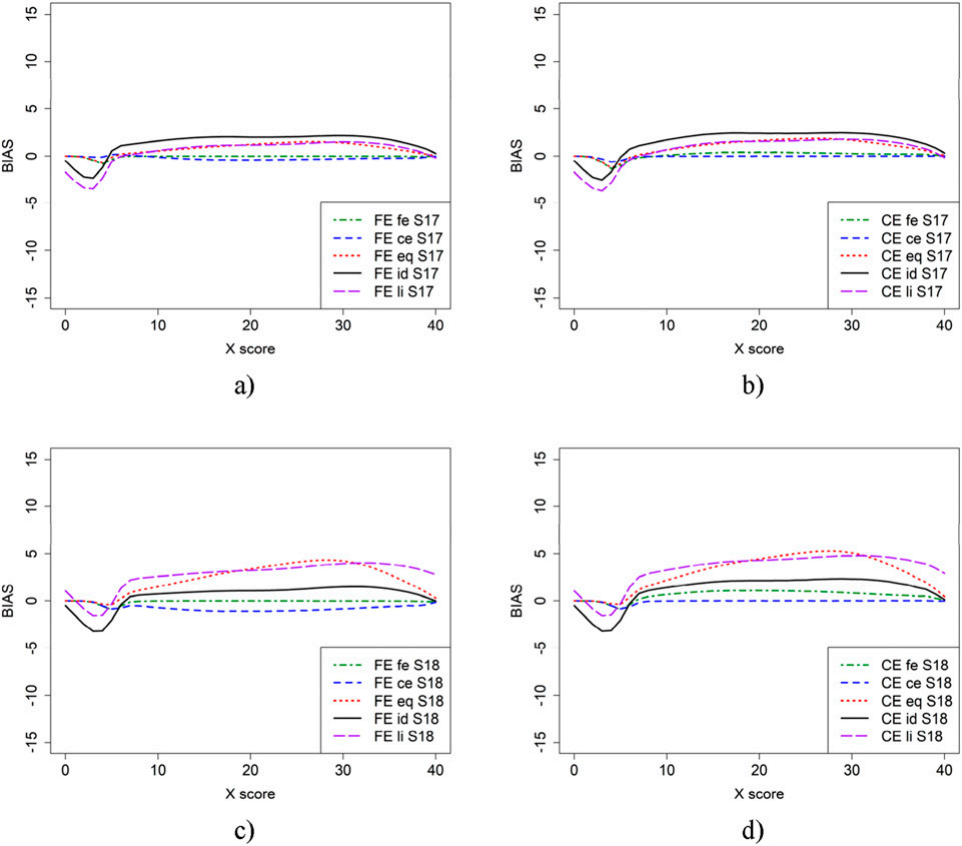
Scenario 17 (a and b) and 18 (c and d), which is similar to scenario 1 and 2 but with shorter regular tests (40 items) and shorter anchor (20 items) test forms.

Figure 6 illustrates the bias results for scenarios where both regular test forms X and Y, as well as the anchor test form, were shorter—40 and 20 items, respectively—in the baseline case (scenario 17) and when population *P* was more able (scenario 18). The results are very different from all previous scenarios, which is probably due to the selection of items in the shorter test forms but also that there are fewer test scores with few test takers compared with when longer test forms were used and very few test takers had higher and lower test scores both in the regular test forms and the anchor test form.

We also studied how a medium-sized correlation (around 0.7) between the regular test forms and the anchor test form impacts bias for groups with similar abilities (Scenario 19) and when population *P* was more able (Scenario 20). Overall, the bias was very similar to the bias results in Figure 2, so we have omitted the figures, but they can be found on the provided github. We further reduced correlation to 0.5 and examined its impact on bias for groups with similar abilities (Scenario 21) and when population *P* was more able (Scenario 22). Since the resulting plots closely resemble those in Figure 2, we have omitted the figures, but they can be found on the provided github.

Summing up, from Figures 2, 3, 4, 5, 6 it is obvious that it does matter which equating method is used together with which criterion function is used to calculate bias. To further examine the 22 scenarios, we examined the mean WAB, as shown in Table 3. Overall, the mean WAB was low when using the same equating method as the criterion function (i.e., FE-fe and CE-ce). The WAB was slightly higher for the combinations of CE and fe, and FE and ce. As expected, the largest WAB values were observed for the eq and id criterion functions. If we compare the other criterion functions to when id is used as the criterion function, we observe that ce, fe and li typically underestimate, while eq overestimates WAB in scenarios 2, 6, 10, 12, 14, 16, 18, 20, and 22, where groups have differing abilities. Interestingly, li produced the same average WAB values as eq for scenarios 12 and 18, which were much higher than when id was used as a criterion function.

**Table 3.** Mean (SD) for Weighted Absolute bias (WAB) Over 500 Replications in the Different scenarios for the two equating Methods (FE and CE) and the Five Criterion Functions.

Criterion function	fe		ce		eq		Id		li	
Equating method	FE	CE	FE	CE	FE	CE	FE	CE	FE	CE
Scenario	Mean (SD)	Mean (SD)	Mean (SD)	Mean (SD)	Mean (SD)	Mean (SD)	Mean (SD)	Mean (SD)	Mean (SD)	Mean (SD)
S1	0.06 (0.02)	1.15 (0.14)	1.13 (0.14)	0.10 (0.02)	1.58 (0.14)	2.64 (0.20)	2.10 (0.17)	3.14 (0.22)	1.74 (0.13)	2.79 (0.19)
S2	0.06 (0.01)	1.21 (0.09)	1.20 (0.09)	0.08 (0.01)	4.40 (0.16)	5.45 (0.18)	2.34 (0.17)	2.39 (0.20)	4.39 (0.16)	5.15 (0.17)
S3	0.06 (0.02)	1.03 (0.12)	1.01 (0.12)	0.10 (0.02)	1.28 (0.12)	2.22 (0.18)	7.44 (0.18)	8.10 (0.22)	2.02 (0.13)	2.69 (0.18)
S4	0.06 (0.01)	1.23 (0.10)	1.23 (0.09)	0.08 (0.01)	4.39 (0.16)	5.49 (0.19)	8.23 (0.19)	7.42 (0.21)	4.38 (0.16)	5.22 (0.18)
S5	0.06 (0.02)	1.18 (0.14)	1.17 (0.14)	0.10 (0.02)	1.54 (0.14)	2.64 (0.20)	4.28 (0.19)	4.19 (0.21)	1.60 (0.12)	2.71 (0.18)
S6	0.06 (0.01)	1.22 (0.09)	1.21 (0.09)	0.08 (0.01)	4.41 (0.16)	5.44 (0.18)	4.01 (0.19)	5.03 (0.21)	4.40 (0.16)	5.29 (0.17)
S7	0.07 (0.03)	1.70 (0.17)	1.68 (0.17)	0.11 (0.03)	2.67 (0.17)	4.23 (0.24)	3.16 (0.19)	4.74 (0.25)	2.76 (0.16)	4.33 (0.22)
S8	0.06 (0.02)	1.34 (0.12)	1.34 (0.12)	0.09 (0.02)	3.15 (0.15)	4.36 (0.19)	3.62 (0.19)	3.76 (0.21)	3.05 (0.16)	3.88 (0.17)
S9	0.06 (0.02)	1.51 (0.15)	1.49 (0.15)	0.11 (0.02)	2.16 (0.15)	3.52 (0.21)	2.66 (0.19)	4.00 (0.23)	2.31 (0.13)	3.69 (0.20)
S10	0.06 (0.02)	1.34 (0.11)	1.34 (0.11)	0.09 (0.01)	3.24 (0.16)	4.46 (0.19)	3.33 (0.19)	3.29 (0.21)	3.17 (0.17)	3.84 (0.17)
S11	0.05 (0.01)	0.79 (0.09)	0.79 (0.09)	0.07 (0.01)	1.44 (0.13)	2.14 (0.13)	1.53 (0.15)	2.29 (0.20)	1.48 (0.12)	2.15 (0.17)
S12	0.07 (0.03)	1.65 (0.13)	1.68 (0.13)	0.07 (0.01)	6.76 (0.19)	8.42 (0.22)	1.61 (0.18)	2.73 (0.23)	6.76 (0.19)	8.41 (0.22)
S13	0.10 (0.03)	1.32 (0.21)	1.28 (0.21)	0.17 (0.04)	1.54 (0.20)	2.77 (0.30)	2.09 (0.24)	3.28 (0.32)	1.73 (0.18)	2.92 (0.28)
S14	0.10 (0.03)	1.29 (0.14)	1.29 (0.14)	0.14 (0.02)	4.39 (0.25)	5.51 (0.29)	2.39 (0.23)	2.47 (0.27)	4.36 (0.25)	5.19 (0.27)
S15	0.17 (0.04)	1.53 (0.31)	1.49 (0.03)	0.30 (0.06)	1.48 (0.27)	2.92 (0.39)	2.07 (0.33)	3.43 (0.44)	1.70 (0.24)	3.07 (0.38)
S16	0.18 (0.05)	1.42 (0.23)	1.43 (0.22)	0.24 (0.04)	4.33 (0.36)	5.58 (0.41)	2.48 (0.36)	2.62 (0.39)	4.29 (0.36)	5.25 (0.37)
S17	0.02 (0.01)	0.35 (0.04)	0.35 (0.04)	0.03 (0.01)	1.14 (0.10)	1.47 (0.12)	1.97 (0.12)	2.29 (0.14)	1.14 (0.10)	1.47 (0.12)
S18	0.03 (0.01)	0.93 (0.05)	0.94 (0.05)	0.03 (0.01)	3.47 (0.01)	4.40 (0.12)	1.18 (0.13)	2.11 (0.14)	3.47 (0.10)	4.40 (0.12)
S19	0.06 (0.02)	1.54 (0.16)	1.52 (0.16)	0.11 (0.02)	1.26 (0.12)	2.64 (0.21)	1.79 (0.18)	3.14 (0.24)	1.41 (0.13)	2.79 (0.21)
S20	0.06 (0.01)	2.03 (0.11)	2.02 (0.11)	0.09 (0.02)	3.62 (0.15)	5.44 (0.18)	2.99 (0.18)	2.40 (0.23)	3.62 (0.15)	5.15 (0.18)
S21	0.06 (0.02)	1.94 (0.19)	1.92 (0.18)	0.12 (0.03)	0.92 (0.11)	2.63 (0.21)	1.44 (0.17)	3.15 (0.25)	1.07 (0.13)	2.79 (0.21)
S22	0.07 (0.02)	3.01 (0.15)	2.99 (0.15)	0.10 (0.02)	2.65 (0.13)	5.44 (0.20)	3.93 (0.18)	2.40 (0.24)	2.64 (0.14)	5.15 (0.19)

fe = frequency estimation, ce = chain equating, eq = equipercentile equating, and id = identity equating, li = linear equating.

## Discussion and Concluding Remarks

The overall aim was to compare the amount of bias in different scenarios when using either CE or FE with five different criterion functions. This research is important because, when comparing different methods across various scenarios, we want the comparison to be fair. An empirical study using real data was also included to illustrate the impact of the choice of criterion function.

From the empirical study it appeared that using fe yielded the lowest bias, regardless of used equating method. The ce criterion function produced varying bias results depending on which equating method was applied. The bias was lower when the li or eq criterion functions were used with the full sample compared with when they were used on the smaller anchor test form sample. A key conclusion from the empirical study is that the method used to calculate bias is important. This finding aligns with Wiberg and González's (2016) overall conclusion that it is crucial to assess equating transformations using multiple approaches. To be able to study different conditions, we proceeded with a simulation study.

From the simulation study, it is evident that the bias is heavily affected by the choice of criterion function. If the equating method and the criterion function is the same—the bias is small in all scenarios. If the equating method and criterion function differ, the bias is larger. This result is important because, to make fair comparisons, we need to choose evaluation tools wisely and possibly use multiple measures as true indicators, a result in line with the conclusions in Wiberg and González (2016) and Leoncio, Battauz, and Wiberg (2022). Using id, li, and eq as the criterion functions instead of fe or ce resulted in general in larger bias across all examined scenarios.

When the groups had differing abilities, bias and WAB were somewhat lower for CE in most cases when id and ce were used as criterion functions, but they were slightly higher for FE compared to when groups were of equal ability. This result is in line with previous studies, which have concluded that CE has been found to produce less bias when group differences are large (e.g., Powers & Kolen, 2014; Wang et al., 2008). Note that our study differs from Wang et al. (2008), who used an internal anchor test form while we used an external anchor test form.

The change in difficulty in one of the regular test forms did not markedly affect bias, except when id was used as a criterion function, which is expected since id assumes that test forms are of equivalent difficulty. However, in the scenario where the groups had similar abilities and the anchor test form was more difficult than the regular test forms, the eq and li criterion functions produced the largest WAB values compared to all other scenarios for both FE and CE. The shorter anchor test had the greatest impact on WAB when the groups had differing abilities and eq and li were used as criterion functions. The decrease in sample size did not significantly impact bias results for most criterion functions. However, reducing the sample size to 500 noticeably increased bias for FE and CE when fe and ce were used as criterion functions, respectively. This is expected, as smaller sample sizes tend in general to increase bias in both FE and CE (Kolen & Brennan, 2014; Livingston & Lewis, 1995; ). A result in line with Kolen and Brennan (2014) was the general conclusion that a lower correlation of 0.5 between the regular test forms and the anchor test form resulted in higher bias and higher WAB for FE and CE when ce and fe were used as criterion functions, respectively. Note, that none of the cited studies in this discussion used different criterion functions when examining bias. Summing up, the overall conclusions are that the choice of criterion function and equating method matters when calculating bias.

There are some limitations with this study. First, we only examined one case involving a shorter anchor test, one case which combined a shorter regular test form and a shorter anchor test form, and we only varied abilities for one group. In the future, it would be interesting to examine more scenarios. Second, we focused on the NEAT design and examined five criterion functions, in the future other data collection designs as well as other criterion functions should be examined.

From a practical perspective, since the amount of bias depends both on the equating method and the chosen criterion function, we recommend using more than one equating method and to use more than one criterion function when calculating bias. Especially if more than one equating method is used, we recommend examining all criterion functions connected to all examined equating methods. Among the examined methods, we recommend using either fe or ce as criterion functions when we have a NEAT design as they in general yielded smaller bias than li, id or eq in the examined cases.
